# Supplementary material for: Genetically diverse mouse models of SARS-CoV-2 infection reproduce clinical variation in type I interferon and cytokine responses in COVID-19
Source: Nat Commun. 2023 Jul 25;14:4481. doi: 10.1038/s41467-023-40076-5 (PMC10368652; doi:10.1038/s41467-023-40076-5)
Supplement: Supplementary file 1 — Supplementary Information [file 41467_2023_40076_MOESM1_ESM.pdf]

Table S1. Summary of percent survival, weight loss, virus burden and BAL cytokine profiles in CC x K18-hACE2 mice.

				Peak weight loss		Average Virus Titers in Lung		Cytokine profile BAL	
Strain (F1)	Sex	%Survival	Mean time to death (days)	%	DPI	3 dpi Mean (Std Dev)	6 dpi Mean (Std Dev)	3 dpi	6 dpi
C57Bl/6 x K18-hACE2	M	14	6	16.7	7	1.3e6 (1.8e6)	7.6e4 (1.3e5)	No/Low	High Group A and Group B
	F	29	7	12.0	7	1.5e6 (1.4e6)	8.2e3 (5.7e3)		
A/J x K18-hACE2	M	33	8	15.0	7	2.8e6 (2.2e6)	3.5e4 (6.1e4)	No/Low	High Group A and Group B
	F	66	UD	6.1	7	2.8e6 (2.6e6)	2.7e4 (2.8e4)		
PWK x K18-hACE2	M	83	UD	-	-	1.6e5 (2.3e5)	BLD	High Group A	Moderate Group A and Group B
	F	83	UD	7.0	7	9.2e4 (1.1e4)	BLD		
NZO x K18-hACE2	M	86	UD	13.9	9	2.2e6 (2.8e6)	3.8e3 (3.7e3)	High Group A	High Group A and B
	F	71	UD	21.9	9	1.4e6 (1.2e6)	1.3e3 (1.0e3)		
129S1 x K18-ACE2	M	86	UD	11.8	7	1.3e6 (7.8e5)	3.0e3 (2.8e3)	High Group A	High Group A and Group B
	F	71	UD	12.0	7	8.1e5 (6.4e4)	2.4e4 (4.0e4)		
CAST/EiJ x K18-ACE2	M	0	6	17.2	7	6.7e6 (7.1e6)	4.2e5(3.5e5)	Low Group A	Low Group A
	F	50	15.5	35.8	8	1.6e6(1.4e6)	3.4e4 (2.9e4)	Low Group A	Low Group A
NOD x K18-ACE2	M	33	6.5	6.0	6	2.5e6 (4.4e6)	4.8e3 (5.3e3)	No/Low	Moderate I and II
	F	83	UD	-	-	5.1e5 (7.9e5)	6.4e3 (8.1e3)	No/Low	Moderate I and II
WSB x K18-ACE2	M	66	UD	32.0	11	4.1e6 (1.8e6)	3.1e4 (1.6e4)	Moderate Group A	High Group A and B
	F	0	8	25.1	8	4.4e6 (4.1e6)	2.5e4 (1.2e4)	Moderate Group A	Moderate Group A and Group B
BALB/c x K18-ACE2	M	100	UD	3.0	5	6.0e5 (5.5e5)	1.6e4 (1.3e4)	No/Low	Low Group B
	F	66	UD	8.3	6	3.7e5 (4.2e5)	6.0e2 (1.2e3)	High Group A	High Group A and Group B
DBA/2 x K18-ACE2	M	57	UD	16.0	10	1.5e6 (1.7e6)	1.7e4 (2.2e4)	No/Low	No/Low
	F	71	UD	3.6	6	1.7e6 (1.2e6)	1.2e3 (1.0e3)	No/Low	High Group A and Group B

UD: undetermined
BLD: below limit of detection

Table S2. Summary of statistical analyses of cytokine and chemokine expression in BAL fluid collected from resistant and sensitive CC x K18-hACE2.

	**Resistant[1] versus Sensitive[2]**		**Kinetics**	
	R>S[3]	S>R[4]	**3 dpi versus 6 dpi[5]**	
	**3 dpi**	**6 dpi**	**Resistant[1]**	**Sensitive[2]**
**Analyte**	**P value**	**P value**	**P value**	**P value**
GM-CSF	8.7e-02	2.4e-01	2.4e-01	**1.3e-03**
IFNalpha	**2.9e-05**	1.8e-01	**1.4e-12**	**7.1e-04**
IFNgamma	**1.1e-03**	5.1e-01	**1.9e-05**	**1.3e-03**
IL1-beta	**2.2e-02**	4.1e-01	**1.7e-02**	**1.3e-03**
IL-12p70	**2.1e-03**	1.0e-01	1.0e-00	**1.2e-02**
IL-13	**1.7e-03**	4.6e-01	**1.2e-03**	**2.2e-04**
IL-18	**1.7e-03**	4.7e-01	**1.2e-04**	**1.2e-04**
IL-2	6.4e-01	6.4e-01	**2.5e-03**	**8.8e-04**
IL-4	5.1e-02	5.2e-01	**7.5e-03**	**1.2e-03**
IL-5	8.8e-01	8.8e-01	**5.7e-03**	**4.2e-03**
IL-6	**1.0e-02**	2.4e-01	**7.7e-06**	8.6e-01
TNFa	**3.9e-03**	2.0e-01	9.1e-02	**2.2e-03**
IL-10	6.7e-01	**1.9e-02**	**1.2e-06**	**1.2e-03**
*IL-17A	**1.7e-02**	5.8e-01	**1.7e-02**	6.8e-01
IL-22	**8.3e-03**	1.0e-01	7.9e-01	5.2e-02
IL-23	8.1e-01	9.4e-01	3.2e-01	3.8e-01
IL-27	**2.9e-03**	8.2e-01	**4.1e-02**	4.7e-01
*IL-9	1.0e-00	1.0e-01	4.6e-01	5.1e-01
Eotaxin	**2.0e-02**	7.2e-01	**3.9e-03**	**7.8e-04**
Gro-alpha/KC	**3.0e-03**	2.1e-01	7.9e-01	1.5e-01
CCL2 (MCP1)	**2.5e-03**	6.1e-01	5.5e-02	1.3e-01
CCL3 (MIP1a)	**2.4e-02**	3.1e-01	5.7e-01	**4.8e-04**
CCL4 (MIP1b)	**1.1e-03**	**4.1e-02**	3.0e-01	**1.1e-03**
CCL5 (RANTES)	7.9e-02	6.2e-01	1.3e-01	**3.7e-03**
CCL7 (MCP3)	**1.5e-02**	8.6e-01	9.3e-01	**4.5e-03**
CXCL2 (MIP2a)	**3.7e-03**	4.2e-01	5.8e-02	6.6e-02
CXCL10 (IP10)	**6.4e-03**	6.3e-01	**6.4e-03**	3.4e-01

*At or below the lower limit of detection.

[1] Resistant mice include PWK x K18-hACE2, NZO x K18-hACE2 and 129S1 x K18-hACE2.

[2] Sensitive mice include K18-hACE2 and A/J x K18-hACE2.

[3] Higher levels of analytes in resistant compared to sensitive

[4] Higher levels of analytes in sensitive compared to resistant

[5] Bold black text indicates statistically significant increase from 3 dpi to 6 dpi. Bold blue text indicates statistically significant decrease from 3 dpi to 6 dpi.

Two-tailed Student *t*-test with 95% confidence interval was used to compare cytokine production in resistant versus sensitive mice (males and females combined) at 3 and 6 dpi. *p<0.05 was considered statistically significant.

Table S3. Summary of statistical analyses of cytokine and chemokine expression in BAL fluid comparing male and female resistant PWK x K18-hACE2 and sensitive K18-hACE2.

	PWK[1] vs B6[2]	PWK vs B6	PWK vs B6	PWK vs B6
	**Females**		**Males**	
	**3 dpi**	**6 dpi**	**3 dpi**	**6 dpi**
**Analyte**	**P value**	**P value**	**P value**	**P value**
GM-CSF	**9.8e-04**	**8.1e-04**	3.4e-01	1.1e-01
IFNalpha	**6.4e-03**	ND	**3.3e-06**	ND
IFNgamma	**5.2e-04**	1.9e-01	**2.8e-03**	3.3e-01
IL1-beta	**6.0e-04**	**4.9e-03**	1.2e-01	2.0e-01
IL-12p70	6.1e-02	**4.9e-02**	**2.7e-03**	4.4e-01
IL-13	**8.9e-06**	**9.8e-03**	9.7e-02	2.4e-01
IL-18	**1.1e-04**	**8.0e-03**	**3.3e-02**	2.0e-01
IL-2	9.7e-02	9.8e-02	9.0e-01	3.1e-01
IL-4	**1.9e-03**	**3.0e-03**	4.1e-01	1.3e-01
IL-5	1.2e-01	**9.6e-03**	9.5e-01	1.6e-01
IL-6	**4.8e-02**	**4.8e-02**	**3.8e-03**	4.4e-01
TNFa	**4.3e-02**	**1.2e-03**	5.4e-02	2.3e-01
IL-10	**5.3e-03**	**2.9e-03**	6.4e-01	1.9e-01
*IL-17A	**2.3e-03**	**1.6e-02**	4.1e-01	6.8e-01
IL-22	**5.2e-03**	9.7e-01	**1.1e-02**	5.2e-01
IL-23	2.1e-01	2.2e-01	3.0e-01	**5.3e-03**
IL-27	6.6e-01	9.7e-01	**8.0e-04**	4.0e-01
*IL-9	7.4e-01	ND	2.2e01	ND
Eotaxin	5.4e-01	**1.3e-03**	8.5e-02	2.0e-01
Gro-alpha/KC	**7.2e-03**	**1.8e-05**	**2.2e-03**	7.6e-02
CCL2 (MCP1)	6.1e-02	**1.4e-02**	**2.2e-03**	3.2e-01
CCL3 (MIP1a)	4.9e-01	**1.8e-02**	**9.8e-03**	7.6e-02
CCL4 (MIP1b)	**6.7e-03**	**1.0e-06**	**1.9e-03**	5.6e-02
CCL5 (RANTES)	8.6e-01	**1.8e-02**	6.0e-02	3.1e-01
CCL7 (MCP3)	**1.3e-02**	**6.5e-03**	8.0e-02	**3.0e-02**
CXCL2 (MIP2a)	**3.6e-03**	1.2e-01	**4.3e-02**	**2.8e-02**
CXCL10 (IP10)	**1.2e-04**	4.9e-01	**3.2e-02**	**7.2e-03**

*At or below the lower limit of detection
[1] Resistant mice include PWK x K18-hACE2
[2] Sensitive mice include K18-hACE2 (C57Bl/6J (B6) background)
Bold black text indicates statistically significance for higher production in PWK than B6.
Bold blue text indicates statistically significance for lower production in PWK than B6.

Two-tailed Student *t*-test with 95% confidence interval was used to compare cytokine production in resistant versus sensitive mice (males and females combined) at 3 and 6 dpi. *p<0.05 was considered statistically significant.

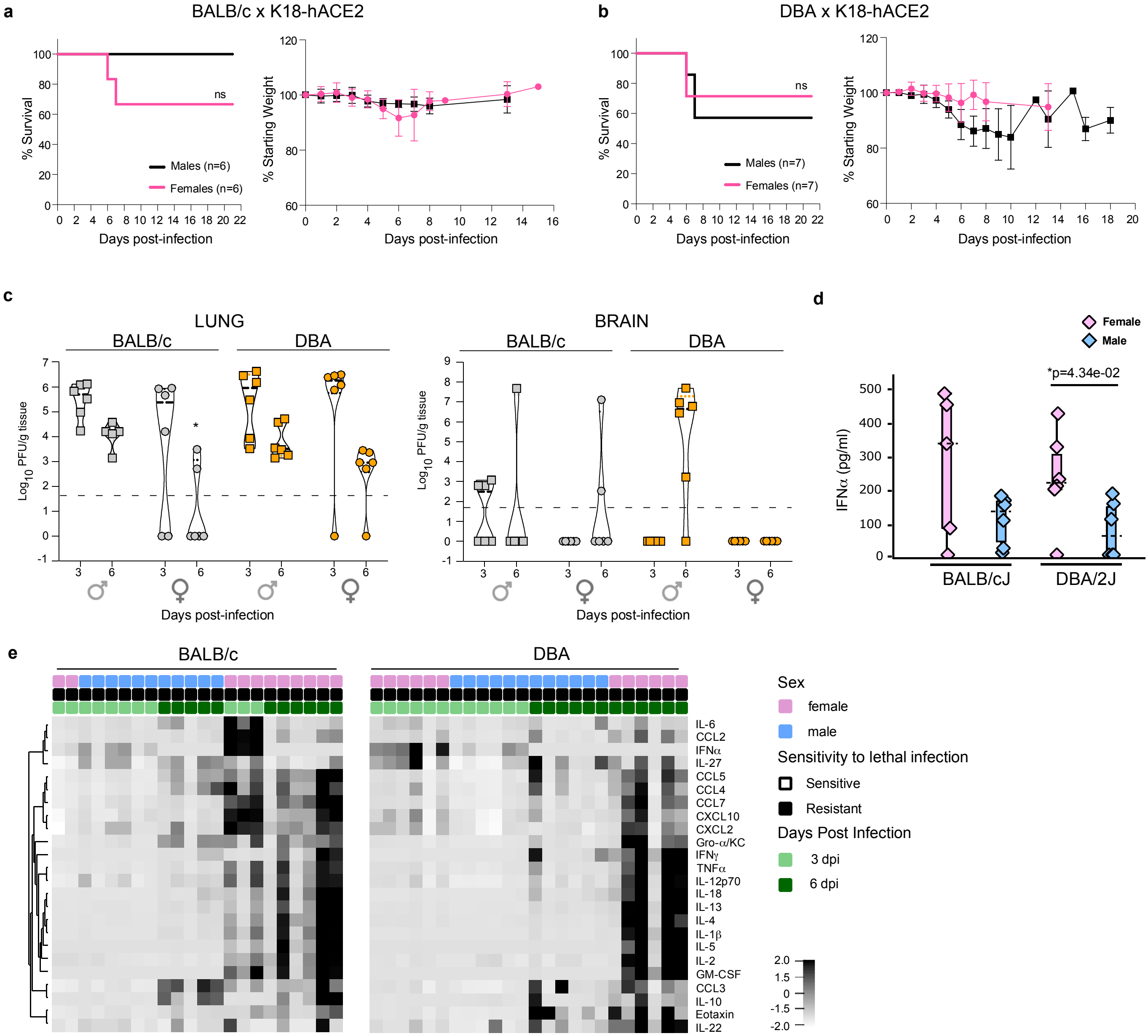

Supplemental Figure 1: Survival, weight loss and SARS-CoV-2 replication kinetics in BALB/cJ x K18-hACE2 or DBA/2J x K18-hACE2 mice. a, b, Survival and weight loss in BALB/cJ x K18-hACE2 (**a**) and DBA/2Jx K18-hACE2 (**b**). Graphs of percent starting weight show the mean ± SD. **c,** Violin plots of virus titers (pfu/g tissue) in lung and brain of male and female mice were measured at 3 and 6 dpi. **d,** IFNα protein in BAL from BALB/cJ and DBA/2J x K18-hACE2 mice at 3 dpi. Box plots show data from individual mice (diamonds), the horizontal dashed lines represent the median value, the box indicates the first and third quartiles, and the whiskers indicate the maxima and minima values (Qlucore software). Two-tailed, unpaired Student's t-test was used to compare male and female responses. *p<0.05 was considered statistically significant. **e,** Heatmap of cytokine expression in BAL. Biological replicates were examined over at least 2 independent experiments and the number of mice assessed per strain (male:female) was as follows: BALB/cJ x K18-hACE2 (6:6); DBA/2Jx K18-hACE2 (7:7) for survival studies. For kinetic measurements of virus load and cytokines n=6 mice/strain/sex/timepoint. Source data are provided as a Source Data file.

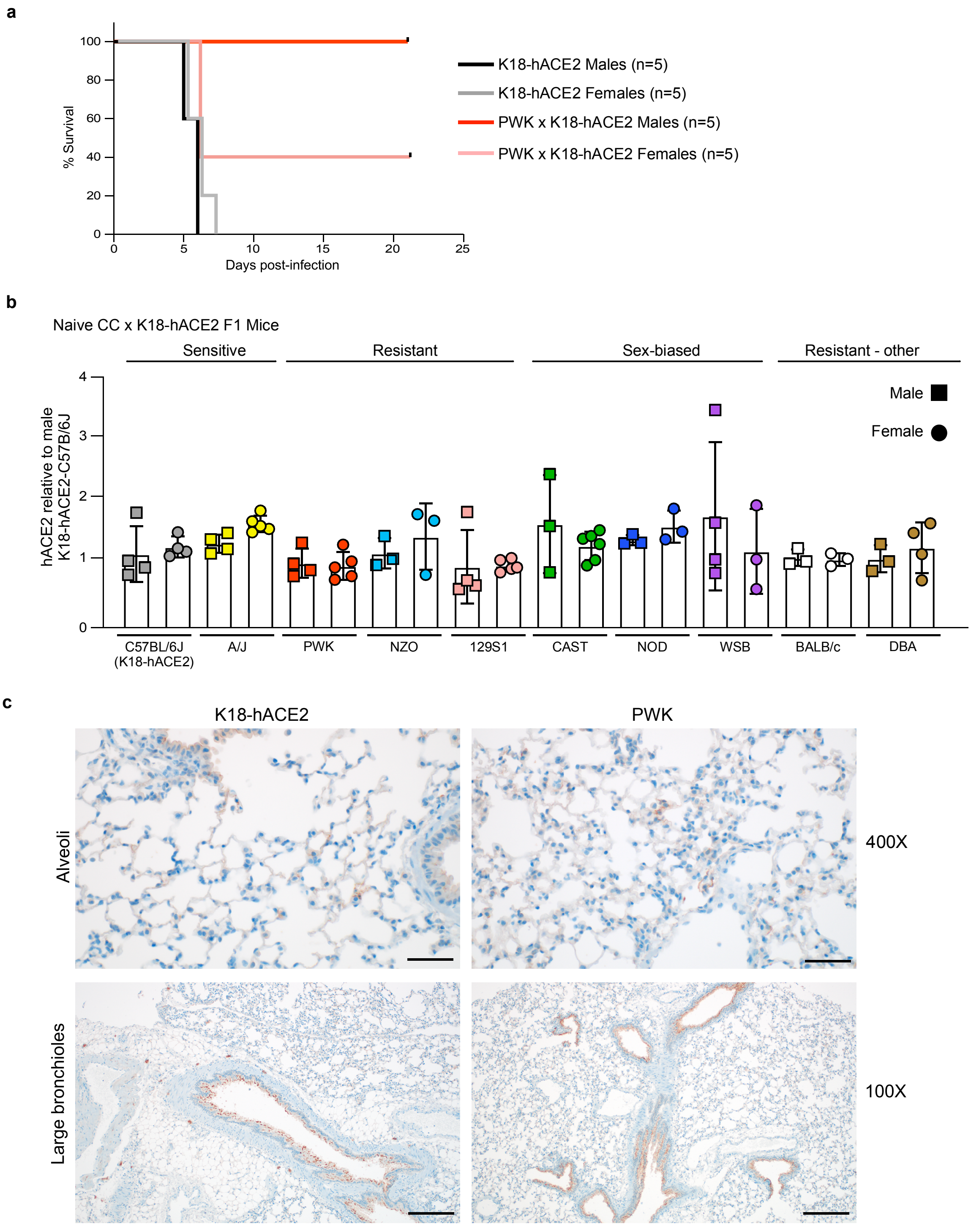

Supplemental Figure 2: Expression of ACE2 in lungs of CC x K18-hACE2 mice. a, Survival curve of K18-hACE2 and PWK x K18-hACE2 male and female mice inoculated intranasally with 10^4 pfu of SARS-CoV-2 and monitored for survival. Biological replicates were examined in one experiment and n=5/strain/sex. **b,** Gene expression specific for *hACE2* was measured by real-time RT-PCR. The graph shows the expression of hACE2 mRNA relative to male K18-hACE2 as the mean ± SD. Biological replicates were assayed in two independent laboratories with similar results and the numbers per strain (male:female) were as follows: (K18-hACE2 (4:4); A/J x K18-hACE2 (4:5); PWK x K18-hACE2 (4:5); NZO x K18-hACE2 (3:3); 129S1 x K18-hACE2 (4:5); CAST x K18-hACE2 (3:6); NOD x K18-hACE2 (3:3); WSB x K18-hACE2 (4:3), BALB/cJ x K18-hACE2 (3:3); DBA/2Jx K18-hACE2 (3:4) **c,** IHC staining for ACE2 in naive K18-hACE2 and PWK x K18-hACE2 mice. Images are representative of 3 mice/sex in K18-hACE2 and PWK x K18-hACE2. Scale bars represent 50 mm (400X); 200 mm (100X).

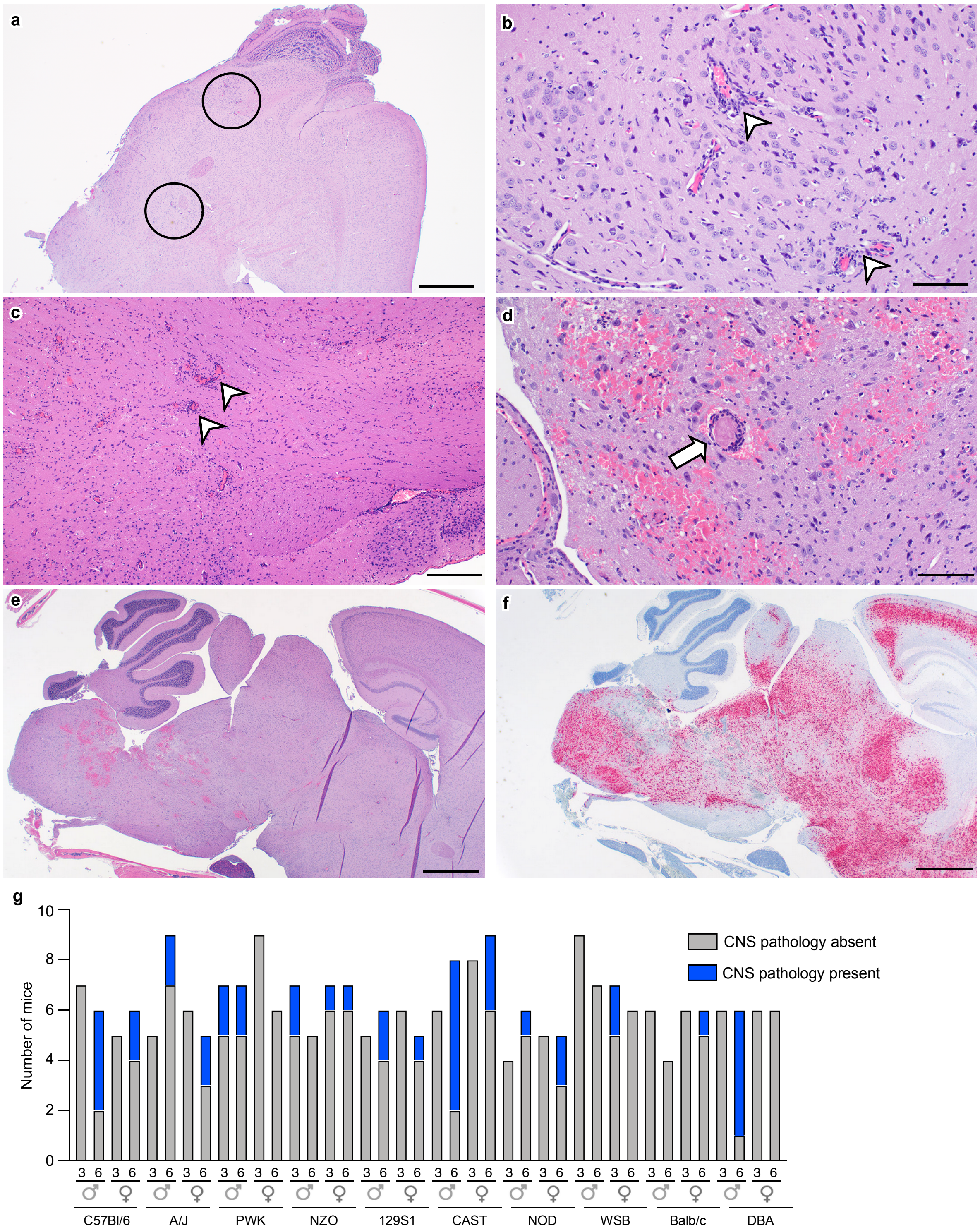

Supplemental Figure 3: Examples of pathology in the brain of SARS-CoV-2-infected CC x K18-hACE2 mice. a, Small focal areas of inflammation (circled) were commonly observed irrespective of genetic strain. Scale bar = 1000 mm. **b,** increased magnification of **(a)** demonstrating perivascular cuffing, gliosis, and evidence of necrotic cells in areas of focal inflammation. Scale bar = 200 mm. **c,** Example of a pronounced lymphocytic inflammatory response with perivascular cuffing and increased cellularity (arrowheads). Scale bar = 100 mm. **d,** Microthrombus occluding cerebral capillaries and hemorrhage (arrow) observed in CAST x K18-hACE2. Scale bar = 100 mm. **e,** Example of extensive hemorrhage in the brain of CAST x K18-hACE2 associated with extensive virus replication as demonstrated in **f**. Microthrombi were observed in 2/8 males and females assessed at 6 dpi. Scale bar = 1000 mm. **f,** serial section relative to **(e)** showing extensive SARS-CoV-2 genome by RNAscope. Scale bar = 1000 mm. **g,** Brain pathology (examples in **a, b** and **c**) was scored as either absent (no evident inflammation) or present and assessed in all biological replicates (male:female) as follows: 3dpi (K18-hACE2 (5:7); A/J x K18-hACE2 (5:5); PWK x K18-hACE2 (7:6); NZO x K18-hACE2 (7:7); 129S1 x K18-hACE2 (6:5); CAST x K18-hACE2 (8:6); NOD x K18-hACE2 (5:4); WSB x K18-hACE2 (6:9), and 6dpi (K18-hACE2 (6:6); A/J x K18-hACE2 (6:7); PWK x K18-hACE2 (6:7); NZO x K18-hACE2 (7:5); 129S1 x K18-hACE2 (5:5); CAST x K18-hACE2 (8:8); NOD x K18-hACE2 (4:6); WSB x K18-hACE2 (5:7).

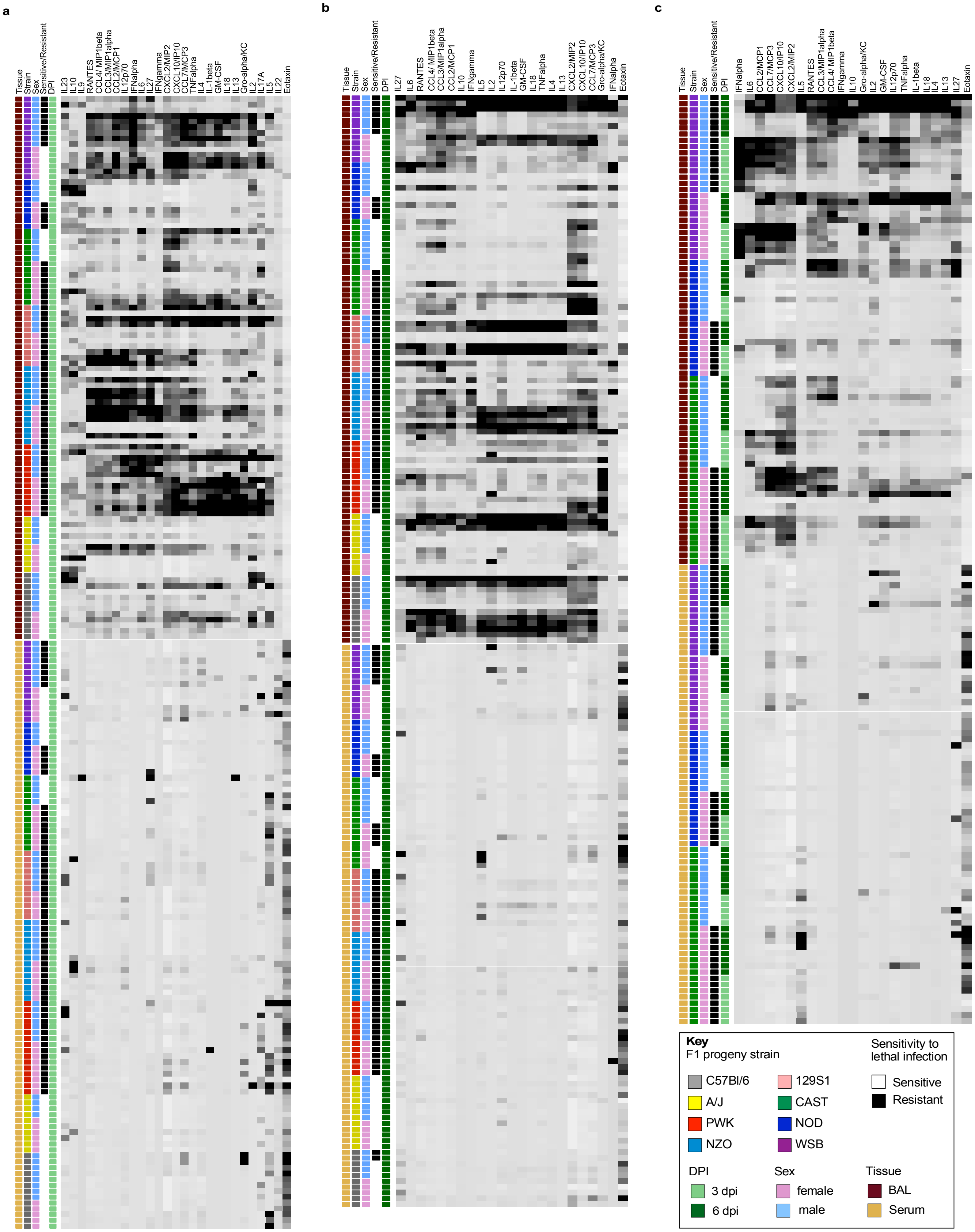

Supplemental Figure 4: Interferon, cytokine and chemokine expression in the serum compared to the BAL fluid in SARS-CoV-2-infected CC x K18-hACE2 mice. Heatmap showing relative cytokine levels, as measured by multiplex cytokine assays, for all eight at CCxK18-hACE2 F1s at **(a)** 3 dpi, and **(b)** 6 dpi, and **(c)** for strains with a sex-bias at 3 and 6 dpi. Source data are provided as a Source Data file.

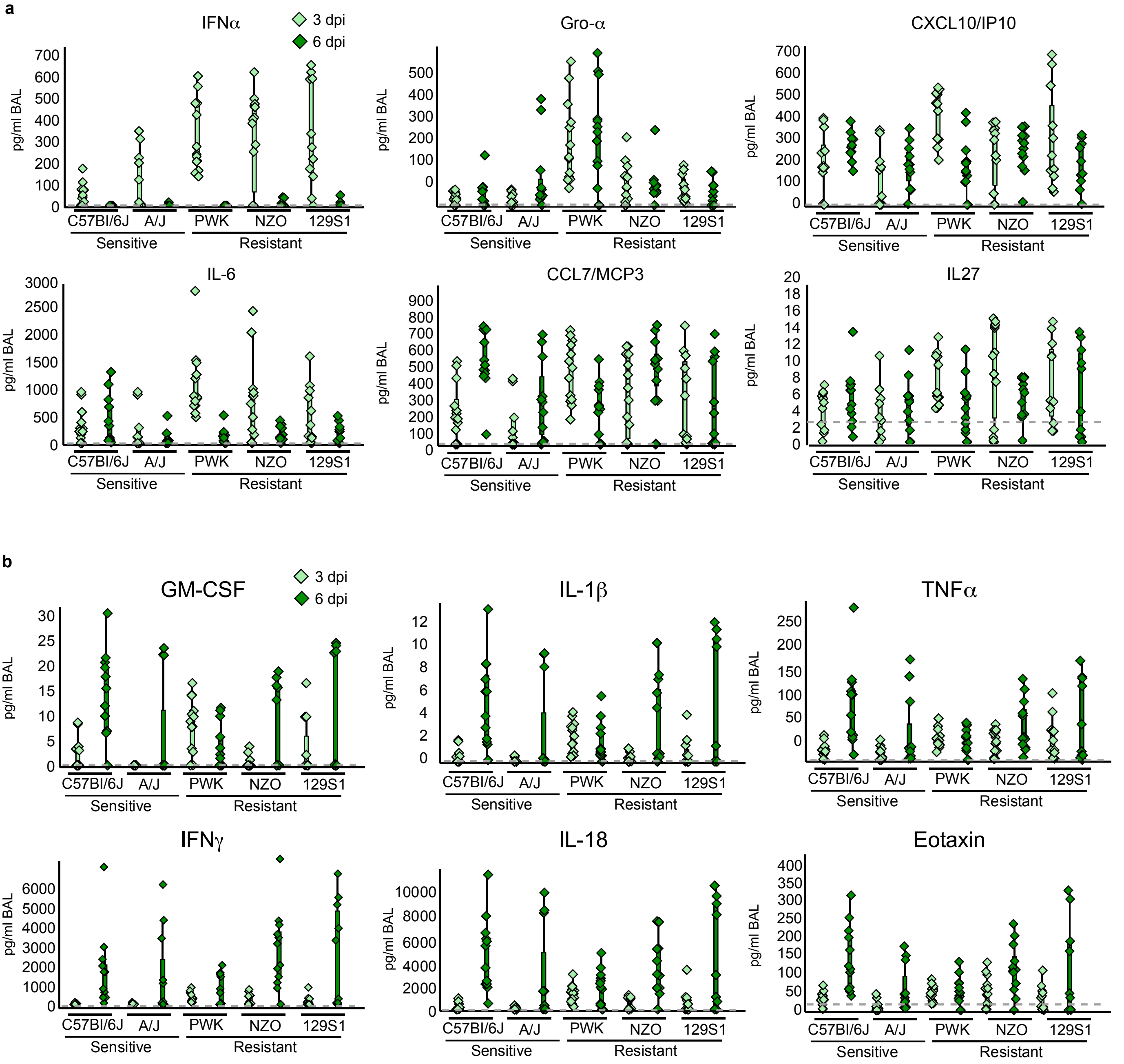

Supplemental Figure 5: Interferon, cytokine, and chemokine expression in the BAL from SARS-CoV-2-infected sensitive and resistant CC x K18-hACE2 mice. a, b, Box plots represent cytokines measured by multiplex cytokine assays in BAL from male and female sensitive (K18-hACE2 and A/J x K18-hACE2) and resistant (PWK, NZO and 129S1J x K18-hACE2) at 3 and 6 dpi. Biological replicates were examined over at least 2 independent experiments and the numbers of mice assessed per strain (male:female) were as follows: 3dpi (K18-hACE2 (5:7); A/J x K18-hACE2 (5:5); PWK x K18-hACE2 (7:6); NZO x K18-hACE2 (7:7); 129S1 x K18-hACE2 (6:5), and 6dpi (K18-hACE2 (6:6); A/J x K18-hACE2 (6:7); PWK x K18-hACE2 (6:7); NZO x K18-hACE2 (7:5); 129S1 x K18-hACE2 (5:5). **a,** “Group A” cytokines with higher production in resistant compared to sensitive CC x K18-hACE2 at 3 dpi. **b**, “Group B” cytokines with delayed and sustained production in sensitive compared to resistant CC x K18-hACE2. The average cytokine expression in BAL of naïve mice is indicated by a grey dashed line. Box plots (drawn in Qlucore software) show individual data points with the box representing the first and third quartiles. Source data are provided as a Source Data file. Statistical comparisons are shown in Table S2.

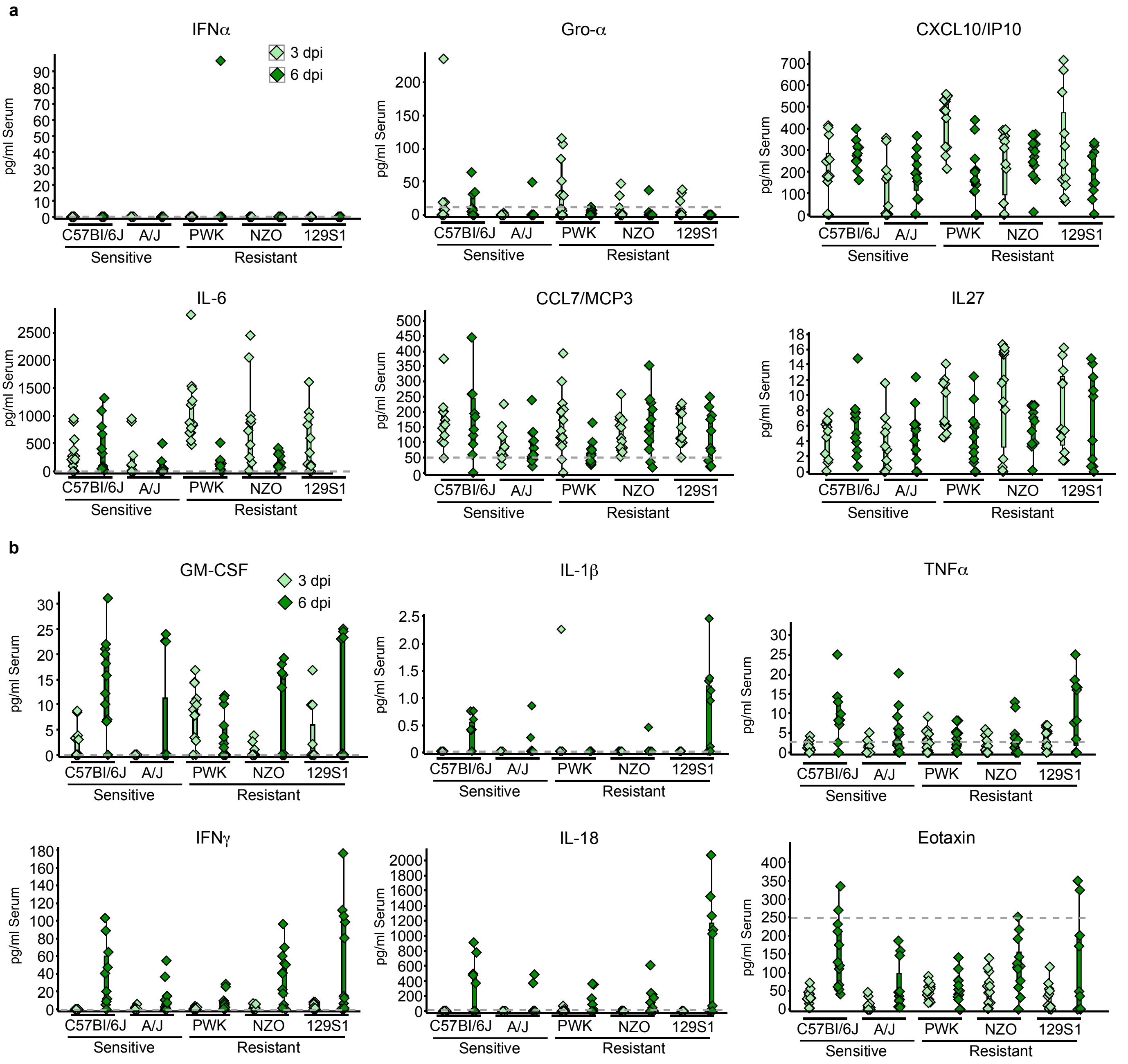

Supplemental Figure 6: Interferon, cytokine, and chemokine expression in the serum from SARS-CoV-2-infected sensitive and resistant CC x K18-hACE2 mice. a, b, Box plots represent cytokines measured by multiplex cytokine assays in serum from male and female sensitive (K18-hACE2 and A/J x K18-hACE2) and resistant (PWK, NZO and 129S1J x K18-hACE2) at 3 and 6 dpi. Biological replicates were examined over at least 2 independent experiments and the numbers of mice assessed per strain (male:female) were as follows: 3dpi (K18-hACE2 (5:7); A/J x K18-hACE2 (5:5); PWK x K18-hACE2 (7:6); NZO x K18-hACE2 (7:7); 129S1 x K18-hACE2 (6:5), and 6dpi (K18-hACE2 (6:6); A/J x K18-hACE2 (6:7); PWK x K18-hACE2 (6:7); NZO x K18-hACE2 (7:5); 129S1 x K18-hACE2 (5:5). **a,** “Group A” cytokines with higher production in resistant compared to sensitive CC x K18-hACE2 at 3 dpi. **b**, “Group B” cytokines with delayed and sustained production in sensitive compared to resistant CC x K18-hACE2. The average cytokine expression in serum of naïve mice is indicated by a grey dashed line. Box plots (drawn in Qlucore software) show individual data points with the box representing the first and third quartiles. Source data are provided as a Source Data file.

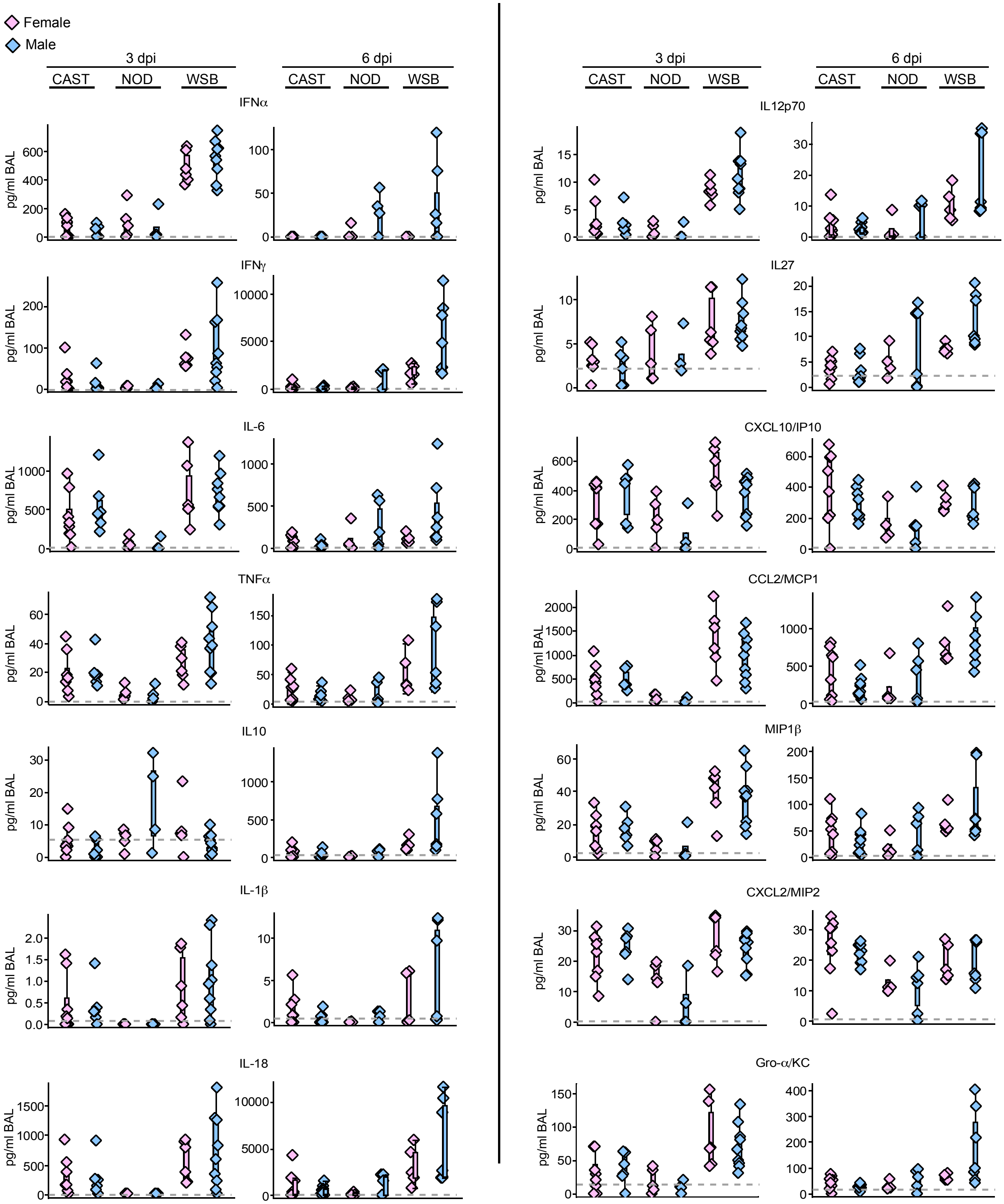

Supplemental Figure 7: Interferon, cytokine, and chemokine expression in the BAL from SARS-CoV-2-infected CC x K18-hACE2 mice with a sex bias. Box plots show cytokine levels in male versus female for CAST, NOD, and WSB x K18-hACE2. Cytokines were measured at 3 and 6 dpi by multiplex cytokine assays. Biological replicates were examined over at least 2 independent experiments and the numbers of mice assessed per strain (male:female) were as follows: 3dpi CAST x K18-hACE2 (8:6); NOD x K18-hACE2 (5:4); WSB x K18-hACE2 (6:9), and 6dpi CAST x K18-hACE2 (8:9); NOD x K18-hACE2 (4:6); WSB x K18-hACE2 (5:7). The average cytokine expression in BAL of naïve mice is indicated by a grey dashed line. Box plots (drawn in Qlucore software) show individual data points with the box representing the first and third quartiles. Source data are provided as a Source Data file. Statistical comparisons are shown in Table S3.
