# Supplementary material for: Genetically diverse mouse models of SARS-CoV-2 infection reproduce clinical variation in type I interferon and cytokine responses in COVID-19
Source: Nat Commun. 2023 Jul 25;14:4481. doi: 10.1038/s41467-023-40076-5 (PMC10368652; doi:10.1038/s41467-023-40076-5)
Supplement: Supplementary file 3 — Reporting Summary [file 41467_2023_40076_MOESM3_ESM.pdf]

nature portfolio

Corresponding author(s): Sonja M Best and Nadia A Rosenthal

Last updated by author(s): Jun 10, 2023

Reporting Summary

Nature Portfolio wishes to improve the reproducibility of the work that we publish. This form provides structure for consistency and transparency in reporting. For further information on Nature Portfolio policies, see our Editorial Policies and the Editorial Policy Checklist.

Statistics

For all statistical analyses, confirm that the following items are present in the figure legend, table legend, main text, or Methods section.

n/a	Confirmed	
☐	☒	The exact sample size (*n*) for each experimental group/condition, given as a discrete number and unit of measurement
☐	☒	A statement on whether measurements were taken from distinct samples or whether the same sample was measured repeatedly
☐	☒	The statistical test(s) used AND whether they are one- or two-sided<br>*Only common tests should be described solely by name; describe more complex techniques in the Methods section.*
☒	☐	A description of all covariates tested
☐	☒	A description of any assumptions or corrections, such as tests of normality and adjustment for multiple comparisons
☐	☒	A full description of the statistical parameters including central tendency (e.g. means) or other basic estimates (e.g. regression coefficient) AND variation (e.g. standard deviation) or associated estimates of uncertainty (e.g. confidence intervals)
☐	☒	For null hypothesis testing, the test statistic (e.g. *F*, *t*, *r*) with confidence intervals, effect sizes, degrees of freedom and *P* value noted<br>*Give P values as exact values whenever suitable.*
☒	☐	For Bayesian analysis, information on the choice of priors and Markov chain Monte Carlo settings
☐	☒	For hierarchical and complex designs, identification of the appropriate level for tests and full reporting of outcomes
☒	☐	Estimates of effect sizes (e.g. Cohen's *d*, Pearson's *r*), indicating how they were calculated

Our web collection on statistics for biologists contains articles on many of the points above.

Software and code

Policy information about availability of computer code

Data collection	BioPlex Manager 6.1.1 software was used for data collection in the multiplex assays.
Data analysis	BioPlex Manager 6.1.1 software was used for data collection in the multiplex assays.<br>graphPad Prism 8.0 (GraphPad Software) was used for statistical analysis and graphing.<br>Qlucore Omics Explorer Version 3.7 (Qlucore AB) was used for statistical analysis and graphing.<br>Spaceranger-1.4.2 pipeline and R studio 4.1.3 with Seurat (v4.0) were used for data analysis of Visium spatial transcriptomics profiling.

For manuscripts utilizing custom algorithms or software that are central to the research but not yet described in published literature, software must be made available to editors and reviewers. We strongly encourage code deposition in a community repository (e.g. GitHub). See the Nature Portfolio guidelines for submitting code & software for further information.

Data

Policy information about availability of data

All manuscripts must include a data availability statement. This statement should provide the following information, where applicable:

- Accession codes, unique identifiers, or web links for publicly available datasets
- A description of any restrictions on data availability
- For clinical datasets or third party data, please ensure that the statement adheres to our policy

All data are available without restrictions. Visium RNA-seq data that support the findings of this study have been deposited in Gene Expression Omnibus (GEO) public database with the accession code GSE231711 (https://www.ncbi.nlm.nih.gov/geo/query/acc.cgi?acc=GSE231711).

Research involving human participants, their data, or biological material

Policy information about studies with human participants or human data. See also policy information about sex, gender (identity/presentation), and sexual orientation and race, ethnicity and racism.

Reporting on sex and gender	N/A
Reporting on race, ethnicity, or other socially relevant groupings	N/A
Population characteristics	N/A
Recruitment	N/A
Ethics oversight	N/A

Note that full information on the approval of the study protocol must also be provided in the manuscript.

Field-specific reporting

Please select the one below that is the best fit for your research. If you are not sure, read the appropriate sections before making your selection.

☒ Life sciences ☐ Behavioural & social sciences ☐ Ecological, evolutionary & environmental sciences

For a reference copy of the document with all sections, see nature.com/documents/nr-reporting-summary-flat.pdf

Life sciences study design

All studies must disclose on these points even when the disclosure is negative.

Sample size	A power analysis was used to determine the sample size number for the animals studies.
Data exclusions	No data was excluded from the analysis
Replication	Animals studies were performed in multiple groups at separate times, such that each strain was assessed in at least 2 independent experiments. Replication attempts were successful. Visium analysis was performed on 3 infected and 2 naive mice per strain.
Randomization	Randomization was not applicable to these studies. Experiments included age-matched mice of both sexes. Animals were housed and handled under the same conditions.
Blinding	Pathologists were blinded to the study sample IDs in their assessment of lung and brain pathology.

Reporting for specific materials, systems and methods

We require information from authors about some types of materials, experimental systems and methods used in many studies. Here, indicate whether each material, system or method listed is relevant to your study. If you are not sure if a list item applies to your research, read the appropriate section before selecting a response.

Materials & experimental systems

n/a	Involved in the study	
	x	Antibodies
	x	Eukaryotic cell lines
x		Palaeontology and archaeology
	x	Animals and other organisms
x		Clinical data
x		Dual use research of concern
x		Plants

Methods

n/a	Involved in the study	
x		ChIP-seq
x		Flow cytometry
x		MRI-based neuroimaging

Antibodies

Antibodies used	ACE-2 antibody (AF933, R&D Systems, lot#HOK0412061); Anti-IFNAR1 (clone MAR1-5A3; cat# BE0241; lot#829122A2) (BioXCell); Mouse IgG1 (clone MOPC-21; cat#BE0083, lot# 785121O1) (BioXCell)
Validation	ACE-2 antibody (AF933) has been extensively used for immunohistochemistry on mouse tissue (https://www.rndsystems.com/products/human-mouse-rat-hamster-ace-2-antibody_af933#product-citations)<br>Anti-IFNAR1 and mouse IgG1 antibodies have been used extensively in in vivo mouse studies including from our own lab https://doi.org/10.1016/j.celrep.2021.109888.

Eukaryotic cell lines

Policy information about cell lines and Sex and Gender in Research

Cell line source(s)	Vero C1008 [Vero 76, clone E6, Vero E6] originally from ECACC (European Collection of Authenticated Cell Cultures), cat#85020206
Authentication	Vero cells were authenticated by the original source (ECACC)
Mycoplasma contamination	Vero cells were confirmed to be negative for mycoplasma contamination.
Commonly misidentified lines (See ICLAC register)	This study did not include commonly misidentified lines.

Animals and other research organisms

Policy information about studies involving animals; ARRIVE guidelines recommended for reporting animal research, and Sex and Gender in Research

Laboratory animals	CC founder x C57BL/6J -K18-hACE2 F1 were provided by The Jackson Laboratories and include the following: B6.Cg-Tg (K18-ACE2)2PrImn/J), 034860; (A/J x B6.Cg-Tg(K18-ACE2)2Prlmn/J)F1/J, 035940; (PWK/PhJ x B6.Cg-Tg(K18-ACE2)2Prlmn/J)F1/J, 035938; (NZO/HlLtJ x B6.Cg-Tg(K18-ACE2)2Prlmn/J)F1/J, 035936; (129S1/SvImJ x B6.Cg-Tg(K18-ACE2)2Prlmn/J)F1/J, 035934; (CAST/EiJ x B6.Cg-Tg(K18-ACE2)2Prlmn/J)F1/J, 035937; (NOD/ShiLtJ x B6.Cg-Tg(K18-ACE2)2Prlmn/J)F1/J, 035935; (WSB/EiJ x B6.Cg-Tg(K18-ACE2)2Prlmn/J)F1/J, 035939; (BALB/cJ x B6.Cg-Tg(K18-ACE2)2Prlmn/J)F1/J, 035941; (DBA/2J x B6.Cg-Tg(K18-ACE2)2Prlmn/J)F1/J 035943. Mice were used at 6-12 weeks of age.
Wild animals	The study did not involve wild animals.
Reporting on sex	Initial characterizations of all strains included males and females. These studies revealed some sex-based findings that are described in the manuscript. IFNAR1 blocking experiments utilized male mice because PWK x K18-hACE2 males produced higher levels of IFN-I than females.
Field-collected samples	This study did not involve samples collected in the field.
Ethics oversight	Animal study protocols were reviewed and approved by the Institutional Animal Care and Use Committee (IACUC) at Rocky Mountain Laboratories (RML), NIAID, NIH in accordance with the recommendations in the Guide for the Care and Use of Laboratory Animals of the NIH.

Note that full information on the approval of the study protocol must also be provided in the manuscript.
